# Nitrogen-Doped Nanoporous Anodic Stainless Steel Foils towards Flexible Supercapacitors

**DOI:** 10.3390/ma15041615

**Published:** 2022-02-21

**Authors:** Wenlei Zhang, Jianle Xu, Gang Li, Kaiying Wang

**Affiliations:** 1 Institute of Energy Innovation, College of Materials Science and Engineering & College of Information and Computer, Taiyuan University of Technology, Taiyuan 030024, China; zhangwenlei@tyut.edu.cn (W.Z.); wangkaiying@tyut.edu.cn (K.W.); 2 Department of Microsystems-IMS, University of South-Eastern Norway, 3184 Horten, Norway

**Keywords:** stainless steel foil, anodic oxidation, nitrogen doping, nanoporous structure, supercapacitors

## Abstract

In this work, we report the fabrication and enhanced supercapacitive performance of nitrogen-doped nanoporous stainless steel foils, which have been prepared by electrochemical anodization and subsequent thermal annealing in ammonia atmosphere. The nanoporous oxide layers are grown on type-304 stainless steel foil with optimal thickness ~11.9 μm. The N-doped sample exhibits high average areal capacitance of 321.3 mF·cm^−2^ at a current density of 1.0 mA·cm^−2^, 3.6 times of increment compared with untreated one. Structural and electrochemical characterizations indicate that the significant enhancement is correlated to the high charge transfer efficiency from nitriding nanosheet products Fe_3_N. Our report here may provide new insight on the development of high-performance, low-cost and binder-free supercapacitor electrodes for flexible and portable electronic device applications with multiple anions.

## 1. Introduction

With rapid growth of global population and high spread of electronic devices, the demanding for energy storage devices and technologies have stimulated high interests towards research and development from academia and industry sectors [1,2]. As the forefront of electrical energy storage system, supercapacitor shows prominent advantages such as fast charging/discharging, high power density, and long cycle life [3], making it promising in the area like portable electronics and hybrid electric vehicles [4]. Among various electrode materials, stainless steel (SS) based devices are receiving more and more attention recently, contributed by its low-cost, binder-free structure, advantageous mechanical property, and reliable conductivity [5].

Although much research has been devoted to the usage of SS served as substrate or collector [6,7,8], little literature is available on the fabrication of SS-based supercapacitor, taking advantages of good supercapacitive performance from Fe_2_O_3_ [9] or Fe_3_O_4_ [10]. Among them, Sagu et al. [11] made wire-like nanoporous structure on the surface of bulk SS by anodic oxidation method, for a capacitance of 18 mF·cm^−2^ in NaOH electrolyte. Deshmukh et al. [12] thermally oxidized type-304 SS mesh in ambient air atmosphere at a temperature up to 800 °C, with a result of 45.92 mF·cm^−2^ average capacitance. Long et al. [13] explored lithium storage and supercapacitive performance of thermally oxidized type-304 SS mesh. However, due to their relatively small specific surface area and limited conductivity, the capacitance value was still far away from applications.

Recently, mixed-anion compounds, conductive polymer compounds or compounding metals with multiple anions beyond the single-oxide ion, offered a new platform for the superior functional materials [14,15,16]. The replacement of oxygen atoms with other anion like nitrogen, sulfur or phosphorus can narrow the bandgap [17], increase vacancies within their crystal structures, and led to high conductivity and good ionic diffusion [18]. Thus, the supercapacitive performance of oxidized SS is willing to be enhanced by the doping process.

Considering the issues raised above, we fabricated an ultra-thick nanoporous oxide layer on the surface of type-304 stainless steel foil by pulsed anodic oxidation method with different applied voltages [19]. A nitrogen doping process was carried out for enhancing capacitance and cycling stability. The samples before and after nitrogen doping have been investigated by structural characterization and electrochemical measurements to deeply understand their correlation among porous structures, constituent components, as well as enhanced electrochemical behaviors.

## 2. Materials and Methods

### 2.1. Synthesis Methods

Nanoporous anodized stainless steel (NASS) sample was synthesized through anodic oxidation method. Prior to oxidation process, the SS foils (type-304, Shanghai Shida Stainless Steel Co., Ltd., Shanghai, China) with dimensions of 10.0 mm × 20.0 mm × 0.1 mm were ultrasonicated in acetone, ethanol, and DI water for 10 min, respectively. The anodic oxidation was performed in a two-electrode electrochemical cell with the SS foil as the working electrode and a graphite plate as the counter electrode. The electrolyte was made by a mixture of 111 g ethylene glycol (C_2_H_6_O_2_ 99.5%, Sinopharm Chemical Reagent Co., Ltd., Shanghai, China), 0.37 g ammonium fluoride (NH_4_F 95.0%, Sinopharm Chemical Reagent Co., Ltd.) and 0.18 g H_2_O. To optimize the supercapacitive performance of NASS, three different potentials, 30, 50 and 70 V were applied with the oxidation time of 2 h at room temperature. The synthesized NASS samples were rinsed in acetone and DI water several times, and then annealed at 500 °C in air for 2 h to stabilize their nanoporous structure. Some NASS samples were further treated under ammonia atmosphere at 500 °C for 1 h to fabricate the nitrogen doped (NASS-N) samples.

### 2.2. Characterization Techniques

The surface morphology of NASS and NASS-N samples were examined using a field emission scanning electron microscopy (FESEM, Hitachi SU8010, Tokyo, Japan). A small amount of powder was scratched from stainless-based samples. The powder was further mixed with ethanol followed by 15 mins’ ultrasonic dispersion, forming a homogeneous solution for further transmission electron microscope (TEM, JEOL JEM-2100F, Tokyo, Japan) observation. The crystal structures were tested by an X-ray diffraction (XRD, Rigaku SmartLab 9, Tokyo, Japan) in the 2θ range of 10–70 degree with CuKα (λ = 1.54 Å) radiation. The chemical analysis was evaluated under an X-ray photoelectron spectroscopy (XPS, Thermo VG Escalab 250Xi, New York, NY, USA). The elemental compositions and species were studied through an energy dispersive spectrometry (EDS, Hitachi SU8010, Tokyo, Japan) equipped in the same SEM.

### 2.3. Electrochemical Measurements

The supercapacitive performance of NASS and NASS-N samples was examined by cyclic voltammetry (CV), electrochemical impedance spectra (EIS) and galvanostatic charge-discharge (GCD) tests employing an electrochemical workstation (Zahner IM6, Kronach, Germany). All the tests were under a standard three-electrode system, where the fabricated samples, platinum mesh and Ag/AgCl were used as working, counter and reference electrode, respectively. 1.0 M Na_2_SO_4_ neutral aqueous solution was served as electrolyte in the electrochemical measurements.

## 3. Results and Discussion

**Figure 1:**
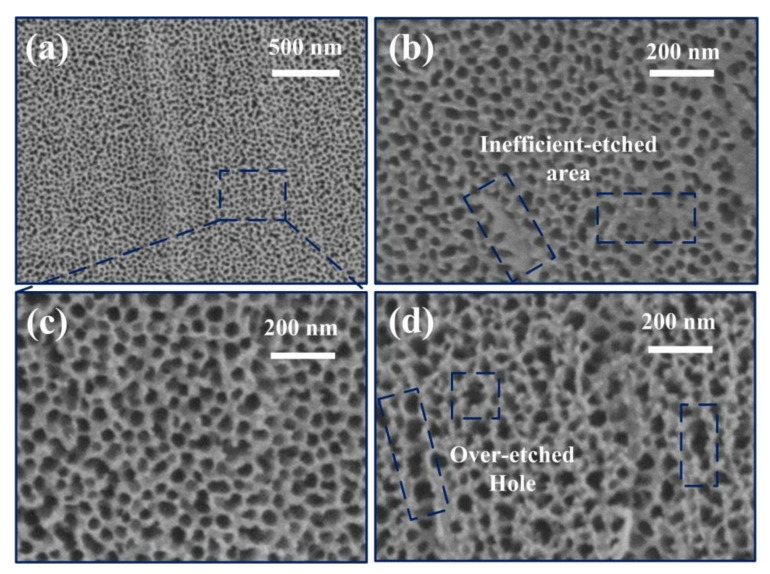
SEM graphs of NASS samples with different anodic oxidation potential. (**a**) The overview graph of NASS sample with applied potential of 50 V. (**b**–**d**) The high magnification graph of NASS sample with applied potential of (**b**) 30 V, (**c**) 50 V and (**d**) 70 V.

Figure 1a shows a typical top-view SEM micrograph of NASS sample. After the anodic oxidation process, a homogenous nanoporous structure was obtained from the surface of SS foil. The lower magnified SEM image is shown in Figure S1 of the Supplementary Materials. According to our previous research, the fabrication process can be explained by the competitive steady state between the oxidation of SS foil and electric field assisted etching of barrier layer, as well as the selective dissolution of Ni oxide in the SS anodic oxidation process [19,20]. Figure 1b–d exhibit the high magnification SEM images of NASS samples with three different anodic potentials. It proved that the pore size can be adjusted through the applied potential during oxidation process. 30 V, 50 V and 70 V oxidized samples had the value of approximate 35, 50 and 55 nm respectively, and increased with the increasing of applied potential. This result had a good agreement with other reports [21,22]. However, higher (70 V) or lower (30 V) potentials may bring some defects, such as the over-etched holes, compact oxide layer or inefficient-etched areas [23], leading to an adverse impact to the supercapacitive performance of NASS.

**Figure 2:**
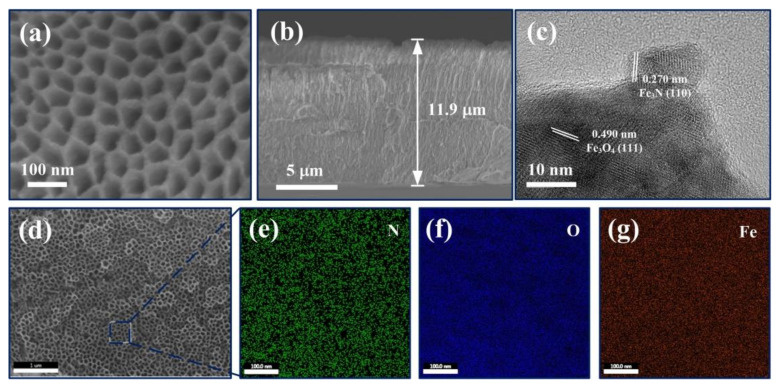
Characterization graphs of NASS-N samples. (**a**) High magnification SEM graph of the sample surface. (**b**) SEM graph of the sample side-wall. (**c**) TEM graph. (**d**–**g**) SEM-EDS graphs.

Figure 2a presents the FESEM graph of 50 V anodic sample after nitrogen doping process in the ammonia atmosphere. The NASS-N sample shows a regular nanoporous structure with the approximate pore size of 50 nm and pore wall thickness of 15 nm. No obvious deformation or addition was found on the structure. Figure 2b shows the cross-sectional view of NASS-N sample. An NASS-N film with thickness ~11.9 μm was successfully fabricated in this work, so far, is the thickest nanoporous Fe-based layer in reported literature, which in principle could offer large surface area and most active materials for electrochemical supercapacitive electrodes [24,25]. TEM was also performed to investigate the microstructure of NASS-N. As shown in Figure 2c, some large nanosheets can be found with the interplanar spacing of 0.49 nm, corresponding to the (111) plane of Fe_3_O_4_ phase (0.489 nm) [26] from the anodic oxidation process of SS foil. The smaller lattice spacing was measured as 0.27 nm, corresponding to the (110) plane of the Fe_3_N phase (0.269 nm) [27]. The result proves that the nitrogen atoms were actually doped into the oxide layer and formed the stable Fe_3_N crystal structure.

**Figure 3:**
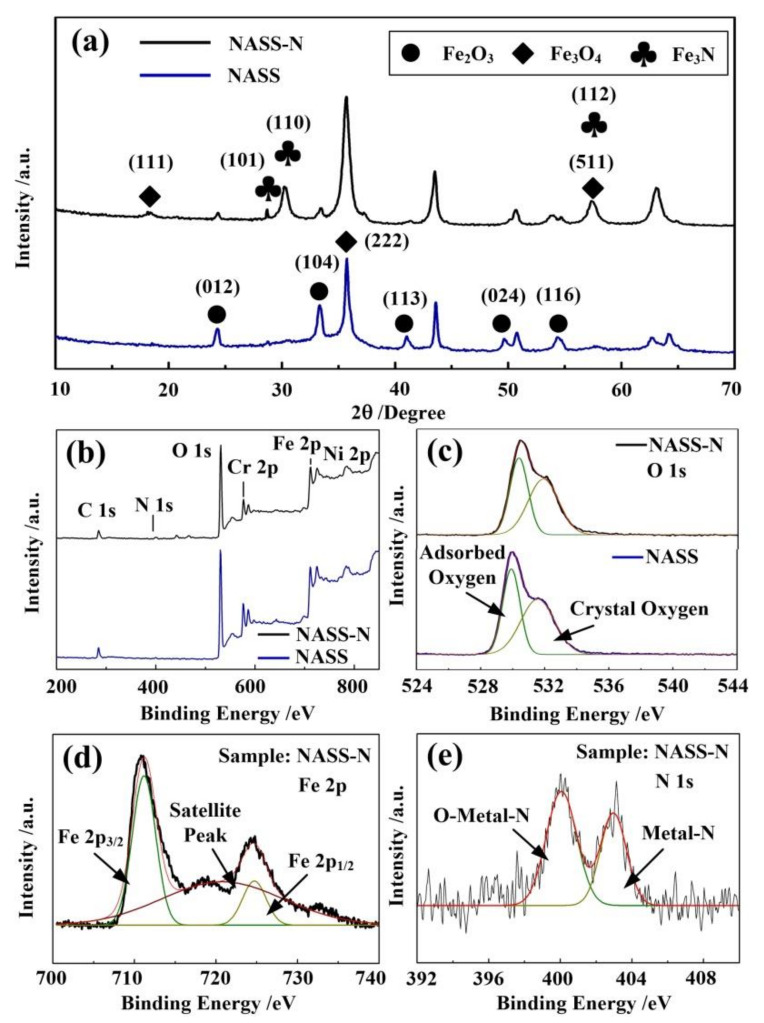
XRD and XPS patterns of NASS and NASS-N samples. (**a**) XRD spectrums. (**b**) XPS survey spectrum. (**c**–**e**) High resolution XPS spectrum of (**c**) O 1s, (**d**) Fe 2p and (**e**) N 1s.

The phase and composition of NASS-N are characterized by SEM-EDS, XRD and XPS. The SEM-EDS elemental mapping of NASS-N is shown in Figure 2d–g, which confirmed that the N, O and Fe were distributed uniformly and homogeneously across the NASS-N sample. Figure 3a presents the XRD patterns of NASS and NASS-N samples. Both of them exhibit characteristic peaks of Fe_2_O_3_ at 24.1° (012), 33.2°(104), 41.2°(113), 49.3° (024) and 54.3° (116) (JCPDS No. 33-0664). After the nitrogen doping process (NASS-N), several new peaks appeared at 28.7°, 30.3° and 57.5°, corresponding to (101), (110) and (112) of Fe_3_N (JCPDS No. 49-1663). These data provide the evidence that some stable Fe_3_N crystals were generated during nitridation process, which shows a good agreement with the observation result from TEM. Moreover, the peak intensity of Fe_3_O_4_ at 18.5° (111), 37.4° (222) and 57.5° (511) (JSPDS No. 75-0449) increased significantly after nitridation. This can be explained as the production of Fe_3_O_4_ from Fe_2_O_3_ by the reduction of ammonia at a relatively high temperature of 500 °C. Further information on the chemical composition and surface electronic state of NASS and NASS-N was acquired from XPS. As shown in Figure 3b, Fe 2p, Cr 2p, C 1s and O 1s peaks in the survey XPS spectrum present the original composition of oxidized SS foil. The relatively weak Ni 2p peak in NASS-N sample was resulted from the selectivity etching of Ni oxides during anodization [19]. A characteristic N 1s peak was observed in NASS-N sample, confirming the successful nitridation process. Figure 3c presents the O 1s spectra of NASS and NASS-N. It can be found that both of the spectra can be divided into two peaks at 530.5 eV and 532.1 eV, corresponding to the adsorbed oxygen and the crystal oxygen respectively. Long et al. [13] discussed that the adsorbed oxygen peak related directly to the defects, and thus increased the conductivity of the samples. Since the intensity ratio between adsorbed oxygen peak and crystal oxygen peak of NASS-N sample (0.85) was larger than that of NASS sample (0.77), its conductivity of NASS-N may also be improved, and then promoted its electrochemical performance. The Fe 2p spectrum of NASS-N sample (Figure 3d) shows two predominant peaks at 711.3 eV (Fe 2p3/2) and 725.3 eV (Fe 2p1/2) with a shake-up satellite peak at 720.1 eV. The former two peaks were the characteristic peaks of Fe^3+^, while the satellite peak suggesting the presence of Fe_3_N after nitrogen doping [28]. This result was also in line with the high-resolution N 1s spectrum of NASS-N sample. As shown in Figure 3e, two peaks with the binding energy of 400.1 eV and 402.9 eV were observed, demonstrating the existence of O-Metal-N and Metal-N, respectively [29]. The above SEM-EDS, XRD and XPS results indicated the successful nitridation process of NASS-N sample collectively.

**Figure 4:**
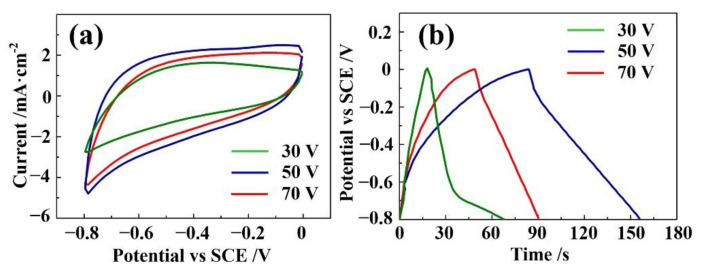
Capacitance performances of NASS samples with different anodic oxidation potential. (**a**) CV curves at scan rate of 100 mV·s^−1^. (**b**) GCD curves at current density 1 mA·cm^−2^.

Electrochemical Measurements were carried out to evaluate the capacitive performance of NASS samples with different anodic fabrication potentials. Figure 4a shows the CV curves of NASS samples anodized at 30 V, 50 V and 70 V with the scan rate of 100 mV·s^−1^, voltage window of −0.8–0 V. The CV curves had a quasi-rectangular shape without distinct redox peak, indicating the electrical double-layer capacitance (EDLC) characteristic of the electrodes [13,30]. Moreover, the 50 V oxidized sample exhibited a larger integrated area and higher current response than the other two, showing the optimized capacitive behavior. Representative GCD curves of these samples obtained at a current density 1 mA·cm^−2^ are presented in Figure 4b. The average areal capacitance of these three samples were 51.3 mF·cm^−2^, 90.0 mF·cm^−2^ and 62.5 mF·cm^−2^ (discharge time was 41 s, 72 s and 50 s respectively) calculated from GCD curves [31]. This result can be explained from the SEM observation results. With lower applied potential, the oxidation process of SS was not enough, leading to the smaller surface area and thinner oxide layer. The lack of active material and small surface decreased its areal capacitance value. However, with higher potential, the oxidation layer was too thick and caused an unexpected drop to the surface conductivity. The charge exchanging process at the surface became not so efficient, leading to an adverse impact to the capacitance. Thus, the 50 V applied potential could be considered as an optimal parameter for achieving suitable active material, surface area and conductivity.

**Figure 5:**
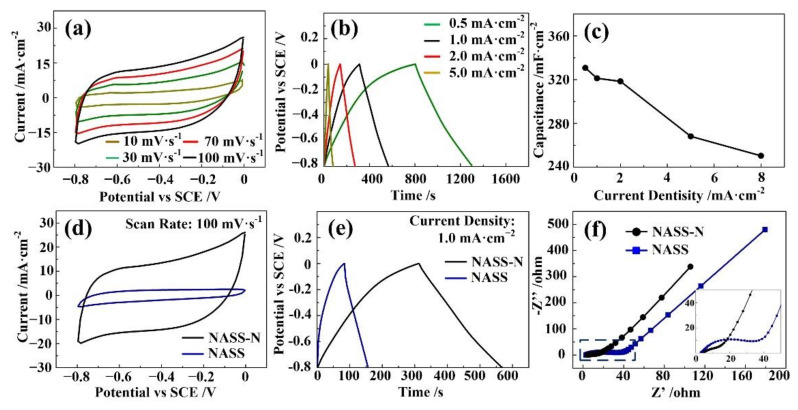
Capacitance performances of NASS-N sample. (**a**) CV curves at different scan rates. (**b**) GCD curves at different current densities. (**c**) Specific capacitances at different current densities. (**d**) CV curves at 100 mV·s^−1^ of NASS-N and NASS samples. (**e**) GCD curves at 1 mA·cm^−2^ of NASS-N and NASS samples. (**f**) Nyquist plots of NASS-N and NASS samples after cycling.

The nitrogen doping processes were performed on the optimal 50 V anodic NASS samples reported above. Figure 5a depicts the CV curves of NASS-N sample at scan rates from 10 to 100 mV·s^−1^. No obvious redox peak was found even at low scan rate, indicating its EDLC characteristic. Furthermore, the good symmetrical characteristic of CV curves proved reliable electrochemical reversibility of NASS-N samples. Figure 5b shows their GCD curves at current density of 0.5, 1.0, 2.0 and 5.0 mA·cm^−2^. The nearly isosceles triangle shape and no obvious potential plateau further confirmed that the EDLC characteristic of NASS-N sample. The areal specific capacitance decreased with increasing current density due to the limited ion diffusion. At a commonly used current density (1.0 mA·cm^−2^) [32,33], the NASS-N sample delivered an average areal capacitance of 321.3 mF·cm^−2^ (discharge time was 257 s). Figure 5c concludes the specific capacitances of NASS-N sample with different current densities. With increasing the current density, the calculated capacitance decreases. However, even the current density is changed from 0.5 mA·cm^−2^ to 8 mA·cm^−2^, the capacitance is dropped from 330.7 mF·cm^−2^ to 250.3 mF·cm^−2^, with 75.7% of the capacitance is retained. The result shows its good rate capability. A series of comparison of electrochemical measurements between NASS and NASS-N sample have been recorded and shown in Figure 5d–f. The CV curve of NASS-N shows much larger integral area than that of NASS sample (Figure 5d), indicating that much higher capacitance was achieved by nitrogen doping process. The detail value was calculated from GCD curves (Figure 5e): NASS-N samples had an average areal capacitance of 321.3 mF·cm^−2^, while NASS-N samples had the value of 90 mF·cm^−2^. The IR drop was also obtained from GCD curves [34]. For NASS samples, the value was relatively high at 0.10 V, resulting from the 11.9 μm-thick anodic oxidation layer. The value decreased to 0.04 V after nitrogen doping process. Figure 5f demonstrates the Nyquist plots of the two samples. Both of them exhibited a quasi-semicircle in the high-frequency region and an inclined line in the low-frequency region. In the low frequency part, the slopes of the straight lines were both larger than 45°, confirming an ideal capacitive behavior of the samples [35]. The NASS-N sample shows a steeper slope than that of NASS sample, indicating smaller Warburg impedance by nitrogen doping [36]. In the high frequency part, a significant decrease was observed in the radius of semicircle for the NASS-N sample, revealing much smaller charge transfer resistance (R_ct_ = 5.23 Ω), which can be contributed due to its improved conductivity by nitrogen doping process. As shown the intercept of the real axis in the high frequency range, both of the samples had relatively small equivalent series resistance of around 3.5 Ω), benefited from the good adhesion of SS substrate with active materials, and their nanopouors structure.

**Figure 6:**
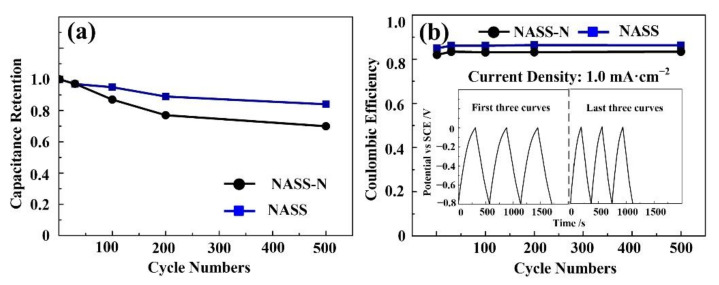
Cyclic performance of NASS-N and NASS samples at 1 mA·cm^−2^. (**a**) Cyclic stability. (**b**) Coulombic efficiency, inset shows the first and last three GCD cycles of NASS-N samples.

Figure 6 shows the cycling performance of the optimal NASS and NASS-N samples by repeating GCD cycles at a current density of 1 mA·cm^−2^. A degradation in cyclic stability was observed by nitrogen doping process. After 500 cycles, 70% of the areal capacitance of NASS-N samples was preserved, while the residual capacitance of NASS samples was more than 84%. The inset in Figure 6b shows the first three and last three GCD cycles, indicating stable and smooth charge and discharge behavior. The coulombic efficiency calculated from the consecutive charge and discharge process (Figure 6b) kept steady, with the value of 85.3% for NASS samples and 82.9% for NASS-N samples. These results agreed with Raut’s [37] research on the similar SS-based supercapacitors.

The NASS sample shows a relatively high capacitance value compared with other report on Fe oxides [11,12,13,38], benefiting from its ultra-thick and unique nanoporous structure. The capacitance of NASS-N sample increases ~3.6 times after nitrogen-doping process through ammonia treatment. This improvement can be benefited from the mixed-anion compounds, especially the formation of Fe_3_N nanosheets in NASS-N sample. In the ammonia atmosphere environment with high temperature, part of Fe_2_O_3_ in the nanoporous structure was reduced to Fe_3_O_4_, and was further transformed to Fe_3_N. Compared with Fe_2_O_3_ or Fe_3_O_4_, Fe_3_N had relatively low molecular weight and can transfer three electrons per formula unit as anode materials [39], and resulted in its high charge transfer efficiency. Moreover, Fe_3_N also has high conductivity and good ionic diffusion due to the vacancies within their crystal structures [18]. Since Fe_3_N nanosheets were planted in the nanoporous structure tightly, the charge carrier mobility of NASS sample can be improved accordingly, leading to smaller impedance in the Nyquist plots, smaller IR drops, as well as larger capacitance value. However, the excess oxygen vacancies caused by nitrogen doping process may lead to the poor cyclic stability [40,41]. The stability of NASS-N sample is still needed for improvement in our future research by the possible methods like carbon shell deposition or glucose carbonization.

## 4. Conclusions

In summary, nanoporous oxide layers have been successfully fabricated on the surface of SS foil through anodization method for supercapacitor applications. The electrochemical anodization potential was optimized and 50 V oxidized sample showed the highest average areal capacitance. High temperature ammonia treatment was adopted for supercapacitive performance enhancement. The optimal thickness of nitrogen doped sample is ~11.9 μm. The morphological and structural characterization such as SEM, TEM, XRD and XPS concluded that Fe_3_N nanosheets were existed in the oxide layer without any damage on its nanoporous structure. The nitrogen doped sample exhibited excellent supercapacitor performance with an areal capacitance as high as 321.3 mF·cm^−2^ at 1.0 mA·cm^−2^, which was 3.6 times than the figure of untreated sample, owing to improved charge transfer efficiency from Fe_3_N nanosheets. The observations and discussions reported in this article may contribute to a better understanding of how to fabricate the high-performance, binder-free SS-based electrodes for supercapacitor usage in the view of constructing multiple anions compounds.

## Data Availability

The data that support the findings of this study are available from the corresponding author upon reasonable request.

## Supplementary Materials

The following supporting information can be downloaded at: https://www.mdpi.com/article/10.3390/ma15041615/s1, Figure S1: lower magnified SEM image of NASS sample.
