# Supplementary material for: Nitrogen-Doped Nanoporous Anodic Stainless Steel Foils towards Flexible Supercapacitors
Source: Materials (Basel). 2022 Feb 21;15(4):1615. doi: 10.3390/ma15041615 (PMC8879062; doi:10.3390/ma15041615)
Supplement: Supplementary file 1 [file materials-15-01615-s001.zip › materials-1540072-supplementary.pdf]

materials

Supplementary

Nitrogen-Doped Nanoporous Anodic Stainless Steel Foils towards Flexible Supercapacitors

Wenlei Zhang [1], Jianle Xu [1,*], Gang Li [1,*] and Kaiying Wang [1,2]

1 Institute of Energy Innovation, College of Materials Science and Engineering & College of Information and Computer, Taiyuan University of Technology, Taiyuan 030024, China; zhangwenlei@tyut.edu.cn (W.Z.); wangkaiying@tyut.edu.cn (K.W.)
2 Department of Microsystems-IMS, University of South-Eastern Norway, 3184 Horten, Norway
* Correspondence: xujianle@tyut.edu.cn (J.X.); ligang02@tyut.edu.cn (G.L.)

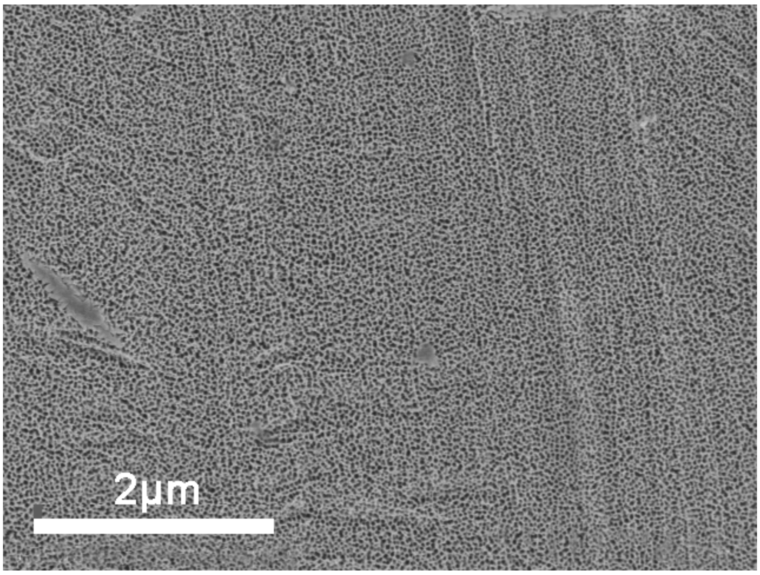

Figure S1. lower magnified SEM image of NASS sample.
